# Effects of polyethylene glycol-grafted phospholipid on the anionic magnetite nanoparticles-induced deformation and poration in giant lipid vesicles

**DOI:** 10.1371/journal.pone.0289087

**Published:** 2023-07-31

**Authors:** Mohammad Abu Sayem Karal, Sharmin Sultana, Md. Masum Billah, Md. Moniruzzaman, Md. Abdul Wadud, R. C. Gosh

**Affiliations:** 1 Department of Physics, Bangladesh University of Engineering and Technology, Dhaka, Bangladesh; 2 Department of Physics, University of Dhaka, Dhaka, Bangladesh; 3 Department of Physics, Jashore University of Science and Technology, Jashore, Bangladesh; Universita Cattolica del Sacro Cuore, ITALY

## Abstract

The hydrophilic polymer polyethylene glycol-grafted phospholipid has been used extensively in the study of artificial vesicles, nanomedicine, and antimicrobial peptides/proteins. In this research, the effects of 1,2-dioleoyl-*sn*-glycero-3-phosphoethanolamine-N- [methoxy (polyethylene glycol)-2000] (abbreviated PEG-DOPE) on the deformation and poration of giant unilamellar vesicles (GUVs)-induced by anionic magnetite nanoparticles (NPs) have been investigated. For this, the size of the NPs used was 18 nm, and their concentration in the physiological solution was 2.00 μg/mL. GUVs were prepared using the natural swelling method comprising 1,2-dioleoyl-*sn*-glycero-3-phosphocholine (DOPC) and PEG-DOPE. The mole% of PEG-DOPE in the membranes were 0, 2, and 5%. The degree of deformation of the GUVs was quantified by the parameter compactness (*C*_om_), which is 1.0 for the spherical-shaped GUVs. The value of *C*_om_ increases with time during the interactions of NPs with GUVs for any concentration of PEG-DOPE, but the rate of increase is significantly influenced by the PEG-DOPE concentration in the membranes. The average compactness increases with the increase of PEG-DOPE%, and after 60 min of NPs interaction, the values of average compactness for 0, 2, and 5% PEG-DOPE were 1.19 ± 0.02, 1.26 ± 0.03 and 1.35 ± 0.05, respectively. The fraction of deformation (*Fr*_d_) also increased with the increase of PEG-DOPE%, and at 60 min, the values of *Fr*_d_ for 0 and 5% PEG-DOPE were 0.47 ± 0.02 and 0.63 ± 0.02, respectively. The fraction of poration (*Fr*_p_) increased with the increase of PEG-DOPE, and at 60 min, the values of *Fr*_p_ for 0 and 5% PEG-DOPE were 0.25 ± 0.02 and 0.48 ± 0.02, respectively. Hence, the presence of PEG-grafted phospholipid in the membranes greatly enhances the anionic magnetite NPs-induced deformation and poration of giant vesicles.

## 1. Introduction

Polyethylene glycol (PEG)-grafted phospholipid is considered to play an important role on the stability of membranes in several ways, including increasing lateral pressure, hydrophilicity of membranes, and affecting the mechanical properties (e.g., bending modulus) of the bilayer [1–3]. As the model of biomembranes (i.e., plasma membrane), artificial membranes comprising of lipids and proteins have been extensively used in biophysical research to understand the processes of living organisms because of their simpler structures and known physical properties [4, 5]. Among the artificial vesicles, giant unilamellar vesicles (GUVs) can be used for the investigations of interaction of nanoparticles (NPs) with membranes containing PEG-grafted phospholipid using optical microscopes because the size-range of GUVs is comparable to that of biological cells [6–8].

NPs of sizes less than 100 nm are considered to be promising biomedical tools having applications in diagnosis and therapeutic tasks because of their versatility and biocompatibility [9, 10]. NPs (both cationic and anionic) are key tools of modern nano-biotechnology [11]. NPs can also cause damage at the plasma membrane upon interaction with the membrane-bound proteins; however, it has been reported that cationic NPs can penetrate the plasma membranes or lipid bilayers better than anionic NPs [12, 13]. Some researchers consider that both cationic and anionic NPs have similar types of effects (e.g., physiochemical mechanisms of interaction) on the charge-neutral bilayer membrane [14].

Currently, NPs are regarded as the auspicious materials on the basis of which it will be possible to create new technologies for the diagnosis and treatment of complex diseases, including cancer, as well as provide a substitute approach to circumvent the issue of multidrug resistance [15–18]. In contrast, adverse effects of the NPs on living organisms, including humans, through biomedical applications (e.g., medical imaging, drug delivery, etc.), have also been detected [19–21]. For instance, the conservational (found in the environment) NPs are prone to pass into the bloodstream as well as spread to supplementary target locations, e.g., liver, heart, etc., through the inhalation process, jeopardizing human health [22–25]. Medical implant materials, forest-fire smoke, MRI contrast agents, ocean spray, pesticides, volcanic activity, food products and packaging, dust storms, vehicle exhausts, etc. are some other possible sources of NPs that have been detected in our human body [26]. In addition, it has been reported that gold NPs can be used effectively for the detection of viruses (the smallest recognized microorganisms) [22, 27]. Hence, the study of the interaction of NPs with biomembranes would be an essential step for the progress of novel technologies in medicine and pharmacology.

Vesicles such as GUVs with diameters ≥10 μm were used for examining and revealing the pore formation event in the membrane due to antimicrobial peptides [28–30], mechanical tension of membrane [31, 32], osmotic pressure effect [33, 34], rupture of membrane comprising polar lipids extracted from *E*. *coli* [35], electroporation [36], and to study the size distribution of self-assemble vesicles [37]. The real-time measurement of the shape and physical properties of a ‘single GUV’ under physiological condition can be performed using optical microscopes. Thus, it is possible to obtain the detail information on the interactions of substances with the GUVs (here, the substance may be a candidate of future antibiotics and the GUV may mimic any pathogenic organism). In the assessment of internal content leakage, such as fluorescent probes, from small or large liposomes/vesicles has been extensively employed to investigate interactions between liposomes and various substances, including drugs, antibacterial agents, and fusogens [38]. The extent of leakage observed serves as an indicator of the substance’s strong interaction with lipid membranes, resulting in the instability of the vesicle and lipid membrane structure. In contrast, the method of suspending liposomes (such as large unilamellar vesicles (LUVs)) does not allow elucidating detail information on the above-mentioned interaction, such as, the primary cause of the leakage from the LUVs, as there may have any of various reasons (e.g., nanopore, local rupture, or complete rupture) behind the leakage event. Hence, the average values of physical parameters for LUV-type liposomes have been obtained from a large number of liposomes, leading to the loss of significant information.

To date, a decent number of research groups have studied the interactions of NPs with lipid bilayer regions of cells/vesicles to elucidate various aspects of the interactions [39–42]. However, the effect of PEG-grafted phospholipid in membranes during the interaction of NPs with the membrane remains unknown. In particular, it is a matter of interest to investigate the interaction of anionic NPs with slight negatively-charged lipid membranes comprising negatively charged PEG-DOPE, and charge-neutral DOPC. In PEG-DOPE structure, DOPE head-group is negatively charged whereas the DOPE-segment has the structural analog of 1,2-dioleoyl-*sn*-glycero-3-phospho- (1′-*rac*-glycerol) (DOPG). Noteworthy, DOPE lipid shows high affinity to form the unstable hexagonal-II phase (H_II_ phase) at an acidic pH of 5–6, but at physiological condition (pH ~7) DOPE helps to form a stable bilayer membrane along with DOPC [43]. Regarding the effect of PEG-lipid in liposomes, it was reported that when the PEG-lipid, DSPE-PEG, was incorporated in DPPC-liposome, fluidity in the interfacial region was greatly enhanced, and the membrane-permeation of fluorescent probe from the liposomes containing PEG-lipid was accelerated significantly [44].

In this research, to investigate the effect of PEG-grafted phospholipid on the interaction of anionic NPs with GUVs comprising PEG-DOPE and DOPC, we used the GUV suspension method. Thus, the constituents of the GUV-membrane were charge-neutral phospholipid DOPC and various mole fractions of negatively charged phospholipid DOPE with grafted PEG. The anionic magnetite NPs of size 18 nm were used in this research. To investigate the effects of PEG-DOPE in the lipid membrane on the interaction of NPs with the lipid membranes, we used DOPC-GUVs with three different mole fractions of PEG-DOPE (i.e., 0, 2 and 5%) in the membranes. The experimental findings are described using some quantitative analyses where some parameters were considered, such as compactness, fraction of deformation, and fraction of poration. Based on the results and quantitative analyses, the possible mechanism has been proposed to explain the phenomena.

## 2. Materials and methods

### 2.1 Materials

1,2-dioleoyl-*sn*-glycero-3-phosphocholine (hereafter abbreviated DOPC) and 1,2-dioleoyl-*sn*-glycero-3-phosphoethanolamine-N- [methoxy (polyethylene glycol)-2000] (hereafter PEG-DOPE) were purchased from Avanti Polar Lipids Inc. (Alabaster, AL, USA). Calcein (Bis[*N*,*N*- bis (carboxymethyl) aminomethyl] fluorescein) and Bovine serum albumin (BSA) were purchased from Sigma-Aldrich (Germany). Diethylene glycol (DEG), Anhydrous Iron (III) chloride (FeCl_3_), Iron (II) chloride tetra-hydrate (FeCl_2_·4H_2_O), sucrose and glucose were purchased from Merck, Germany.

### 2.2 Preparation and purification of PEG-DOPE/DOPC-GUVs

**Fig 1:**
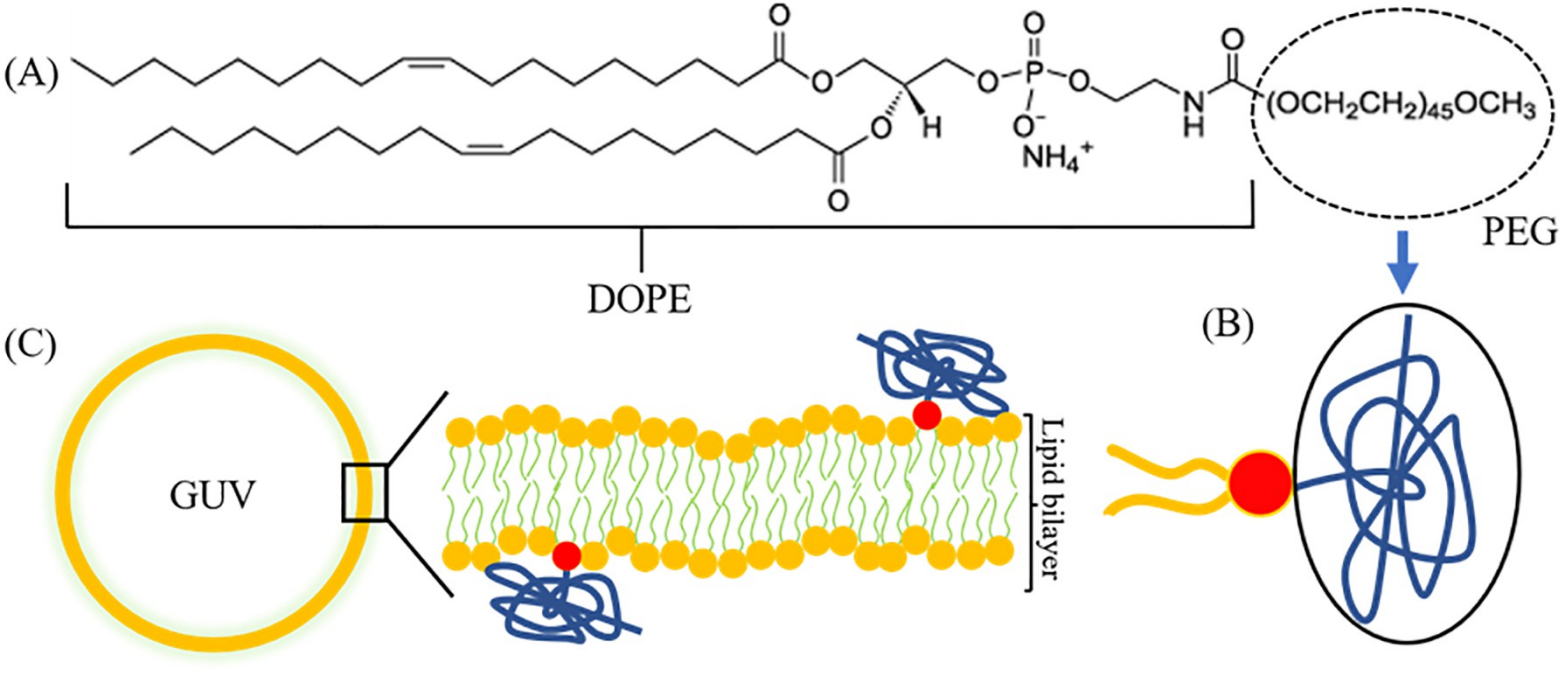
Structure of PEG-DOPE and the schematic diagram of a PEG-DOPE/DOPC-GUV. (A) Structure of PEG-DOPE highlighting the PEG polymer. (B) Cartoon depiction of a PEG-DOPE molecule. (C) Schematic diagram of a PEG-DOPE/DOPC-GUV with the magnification of a membrane-segment that depicts the model arrangement of DOPC (yellow-head groups) and PEG-DOPE (red-head groups) lipid molecules in the bilayer. The long chain (arbitrarily twisted threads in blue) of PEG polymer is attached to the DOPE headgroup.

PEG-DOPE/DOPC-GUVs were prepared by the natural swelling method in MilliQ water [45]. Fig 1 shows the schematic view of a GUV with the cartoon depiction of PEG-DOPE lipid. To prepare the GUVs, at first, 200 μL of PEG-DOPE/DOPC (0/100) or PEG-DOPE/DOPC (2/98) or PEG-DOPE/DOPC (5/95) [here, 0, 2, and 5 indicate the molar ratio] were taken in a 4.5 mL glass vial. That is, the corresponding mole% of PEG-DOPE in the lipid mixture were 0, 2 and 5, respectively. Then, the lipid mixture in chloroform was dried with a gentle breeze of nitrogen gas through a pasteur pipette to produce a homogeneous lipid film on the bottom part of each glass vial. The vials were then kept in the vacuum desiccator for ≥12 h. Next, a small amount of MilliQ water (20 μL/vial) was added to the dried lipid films in each vial and incubated at 45–47°C by dipping the vials in a hot water bath (pre-hydration) for about 8 min.

Later, the samples were incubated with 1 mL/vial MilliQ water containing 0.10 M sucrose for 2.5 h at 37°C. For the purification of GUVs through the removal of smaller GUVs (<10 μm) and LUVs, membrane filtering method was used [46] which provided the comparable outcomes reported recently by us [47]. After the purification, 200 μL purified GUVs suspension (the internal solution of the GUVs contains 0.10 M sucrose in MilliQ (also contains 1.0 mM calcein in the case of leakage experiment); the external solution of the GUVs contains 0.10 M glucose in MilliQ) was transferred into each microchamber that was coated earlier with 0.10% (w/v) BSA in the same solution containing 0.10 M glucose for 30 min. Sugar asymmetry was created between the inside and the outside of GUVs to visualize the GUVs in the suspension. Due to the lower density of the glucose solution (outside environment of the GUVs) compared to that of sucrose solution (inside environment of the GUVs), GUVs were settled down on the bottom surface of the microchamber.

### 2.3 Synthesis of magnetite NPs and dissolution into the solution

**Fig 2:**
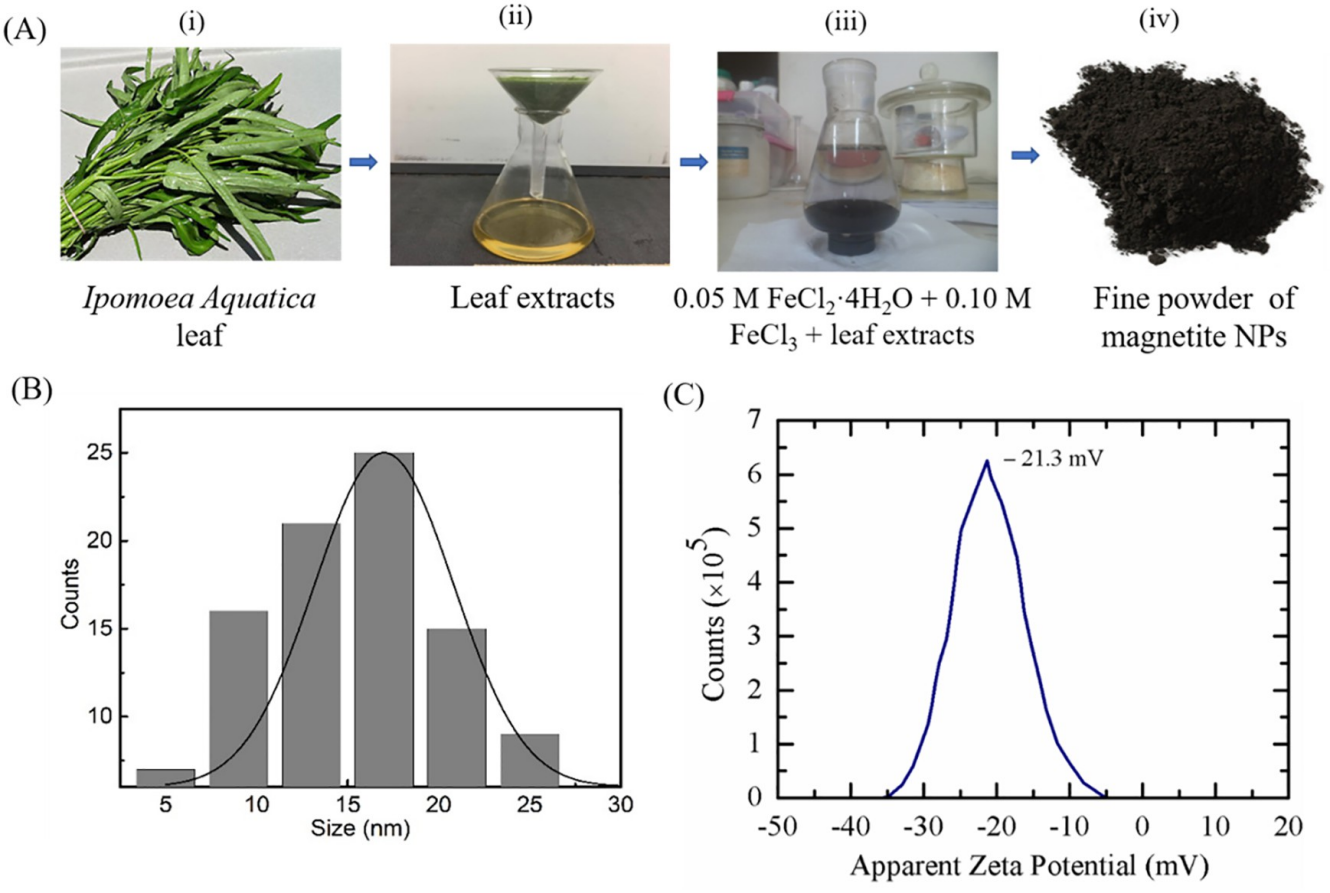
Various steps of synthesis of *Ipomoea aquatica* leaf extracts mediated NPs and their characterizations. (A) Sequence of the synthesis processes is presented through steps (i) to (iv). (B) Particle size distribution of NPs depicting most of the NPs are in the size range of 15–20 nm and overall counts follow Gaussian distribution. (C) Determination of the apparent Zeta potential of the synthesized NPs in deionized water to obtain their charge nature. (B) is reprinted with permission [48] and (C) is reprinted with permission [49].

Anhydrous Iron (III) chloride (FeCl_3_) and Iron (II) chloride tetrahydrate (FeCl_2_·4H_2_O) were employed as precursors in the simple green synthesis process to prepare spinel magnetite (Fe_3_O_4_) NPs, while *Ipomoea aquatica* (water spinach) leaf extracts were used as a source of reducing and capping agents. The step-wise illustration of the entire synthesis process is shown in Fig 2. The synthesized NPs were characterized by gas chromatography-mass spectrometry (GC-MS), fourier transform infrared (FTIR), and energy dispersive X-ray (EDX) to assure the appropriate incorporation of leaf extracts biomolecules in the NPs. The detailed synthesis processes and characterizations were reported previously [48, 49]. A brief description to synthesize the NPs is provided here.

*Ipomoea Aquatica* leaves, which were available in Bangladesh, were purchased from the local market.

The leaves were washed using MilliQ water, as depicted in Fig 2(A-i). After washing, the leaves were dried in an oven at 60°C. Once dried, the leaves were processed into a paste using a blender. Approximately 30 g of the paste was taken and mixed with 100 mL of MilliQ water. The mixture was heated to 80°C and stirred at a speed of 800 rpm for a duration of 4 hours. Following the heating and stirring process, the leaf extracts were filtered using a filter paper, as shown in Fig 2(A-ii). The filtered solution containing the *Ipomoea Aquatica* leaf extract was collected for further use or analysis. At first, 10 mL of 0.05 M FeCl_2_·4H_2_O and 10 mL of 0.10 M FeCl_3_ were stirred at 800 rpm for 10 min keeping the temperature at 60°C, followed by 5 mL leaf extracts added (Fig 2(A-iii)). After 10 min of mixing, 10 mL of 0.10 M NaOH was added. During the process of adding NaOH to the solution, the NPs underwent precipitation, causing them to settle at the bottom. The reaction scheme illustrating this process is as follows:

$$\begin{array}{c} Ipomoea\;aquatica+{\text{FeCl}}_{2}\cdot 4{\text{H}}_{2}\left(\text{aq}\right)+2{\text{FeCl}}_{3}\left(\text{aq}\right)+8\text{NaOH}\left(\text{aq}\right)\\ \to \left[Ipomoea\;aquatica/{\text{Fe}}_{3}{\text{O}}_{4}\right]↓\left(\text{s}\right)+8\text{NaCl}+8{\text{H}}_{2}\text{O}\left(\text{aq}\right) \end{array}$$

The NPs were precipitated at the bottom of a glass beaker, and then collected using a bar magnet. These NPs were dried for a couple of days at 60°C, and then grinded for fine powder (Fig 2(A-iv)). The size distribution of the NPs is shown in Fig 2(B), which was obtained from dynamic light scattering (DLS). The average size of NPs was 18.0 ± 0.5 nm (here ± indicates SErr). The zeta potential of the synthesized NPs was −21.3 ± 4.8 mV (here ± indicates SD) (Fig 2(C)), indicating anionic in nature. A series of experiments was carried out using the specified combinations, and consistent results were found. The selection of these particular values was based on prior evidence and the expected outcomes derived from the employed synthesis method. These combinations were determined to be suitable for achieving the desired results in the experiments, taking into account established knowledge and previous findings.

The initial solution was prepared by dissolving NPs at 0.025 mg/mL in a mixture consisting of 76% diethylene glycol (DEG) and 24% MilliQ water in glucose. Next, the solution was subjected to sonication for a duration of 30 minutes to ensure proper dissolution of the NPs. The initial solution was diluted in MilliQ water containing glucose, resulting in a 0.006 mg/mL NPs solution. This new solution contained 1.8% DEG, 98.2% MilliQ. Subsequently, 100 μL of the 0.006 mg/mL NPs was mixed with 200 μL of GUVs suspension in the microchamber. Within several minutes, the NPs concentration in the entire suspension of GUVs reached the equilibrium. Thus, the final concentration of NPs in the microchamber was 2.00 μg/mL in glucose solution. Noteworthy, the NPs solution contained a negligible percentage of DEG (~ 0.6%) in glucose solution.

### 2.4 Investigation of the deformation of PEG-DOPE/DOPC-GUVs induced by 2.00 μg/mL NPs

The deformation of GUVs with various mole% of PEG-DOPE due to the interaction of 2.00 μg/mL NPs was investigated. GUVs were observed using an inverted phase-contrast fluorescence microscope (Olympus IX-73, Japan) with a 20× objective at 25 ± 1°C and at 50–55% relative humidity. The deformation of the GUVs was monitored and recorded using a charge-coupled device (CCD) camera (Olympus DP22, Japan) connected with microscope. The recording speed of the camera was 25 frames per second. To quantify the deformation of the GUVs at any instant of time, the measurable parameter is necessary to consider, such as, the ‘compactness (*C*_om_)’, which has been broadly used in contemporary bioscience imaging processes, defined as follows [50]:

$$C_{om}=\frac{P^{2}}{4\;\pi \;S_{cr}}$$ (1)

where, *P* and *S*_cr_ are the perimeter of the GUV and area of cross-section of the GUV (that is, area of the 2D view of the GUV observed using optical microscope), respectively. Thus, compactness is a dimensionless parameter whose value is minimum (= 1.0) for a spherical-shaped GUV. The quantity *C*_om_ rises with the deformation of GUV from spherical one. The recorded images were analyzed using MATLAB image processing tool. The compactness measurement only considers a 2D projection of a 3D shape, and so does not capture the full extent of a 3D deformation (unless the deformation occurs entirely in the plane of the image). The procedure to measure the *C*_om_ is described in the supplementary information (SI 1 in S1 File). A GUV is considered to be deformed when its *C*_om_ value exceeds 1.0.

### 2.5 Encapsulating calcein leakage from PEG-DOPE/DOPC-GUVs-GUVs induced by 2.00 μg/mL NPs

To study the pore formation of GUVs induced by NPs, 100 μL of NPs were introduced to a 200 μL suspension of PEG-DOPE/DOPC-GUVs containing 1.0 mM calcein (a water-soluble fluorescent dye with high quantum yield) was used in the GUV lumen to monitor the time-course of fluorescence of the GUV during the interaction with NPs [51]. Like the experiment of deformation of the GUVs, here we also varied the concentration of PEG-DOPE in the GUV-membrane accordingly to examine the effect of PEG-DOPE on NPs-induced pore formation in GUVs. The pore formation in the membrane was recognized by the leakage out of calcein probe from the GUV-lumen. Two neutral-density (ND) filters (25ND6, 25ND25) were used in the fluorescence microscope for avoiding the photobleaching effect of calcein. The microscope objective, stage temperature, relative humidity, recording speed was kept the same as mentioned in section 2.4.

### 2.6 NPs-induced fraction of deformation (*Fr*_d_) and fraction of poration (*Fr*_p_) of GUVs

We examined the NPs-induced deformation and poration probabilities of GUVs in different microchambers. A photo of microchamber used for the observation of GUVs is shown in SI 2 Fig in S1 File. The fraction of deformation (*Fr*_d_) is used to represent the probability of deformation among the observed GUVs, while the fraction of poration (*Fr*_p_) represents the probability of poration. To study the *Fr*_d_ and *Fr*_p_ induced by NPs, 100 μL of NPs were introduced to a 200 μL suspension of PEG-DOPE/DOPC-GUVs. The final concentration of NPs in the suspension of vesicles was 2.00 μg/mL (see section 2.3). The GUVs were observed using a phase contrast microscope, with the focus unchanged throughout the 60-minutes recording period. The observations were made at various time points: 0, 10, 20, 30, 40, 50, and 60 minutes. For each PEG-DOPE, 3–5 independent experiments were conducted, with each experiment consisting of approximately 60–80 GUVs. At each time point, the *Fr*_d_ was calculated by dividing the number of deformed GUVs (*N*_d_) by the total number of observed GUVs (*N*_t_). Similarly, the average *Fr*_d_ values with standard error (SErr) were calculated for each time point across the 3–5 independent experiments. Additionally, the *Fr*_p_ is determined by dividing the number of porated GUVs (*N*_p_) by the total number of observed GUVs (*N*_t_). The average *Fr*_p_ values with SErr were calculated in a similar manner to the calculation of *Fr*_d_. Thus, it has been quantified the probabilities of deformation and poration in GUVs under the given experimental conditions, providing insights into their behavior and response to the microchamber environment.

## 3. Results

### 3.1 Deformation of PEG-DOPE/DOPC-GUVs induced by NPs under various concentrations of PEG-DOPE

**Fig 3:**
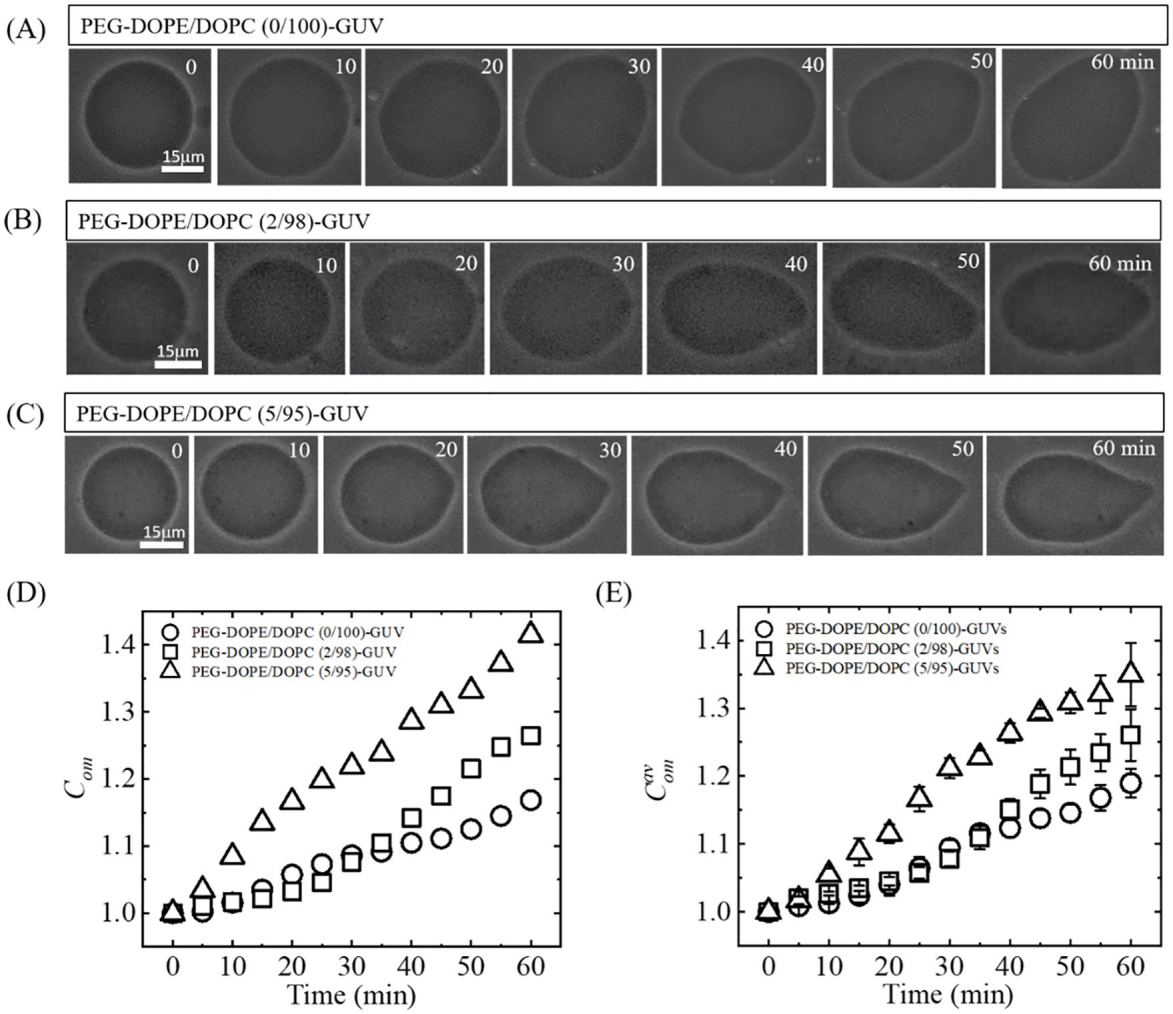
Deformation and compactness of PEG-DOPE/DOPC-GUVs induced by 2.00 μg/mL NPs under various mole% of PEG-DOPE. (A), (B), (C): Typical phase-contrast images of deformation of the PEG-DOPE/DOPC (0/100)-GUV, PEG-DOPE/DOPC (2/98)-GUV, and PEG-DOPE/DOPC (5/95)-GUV with the advancement of interaction time. (D) Time-course of compactness (*C*_om_) of the corresponding GUVs as shown in (A, B & C). (E) Time-course of the average compactness ($C_{om}^{av}$) of the examined GUVs.

To elucidate the effect of PEG-DOPE on the NPs-induced deformation (i.e., shape change) of the PEG-DOPE/DOPC-GUVs, we investigated the interaction of NPs with the GUVs under various mole% of PEG-DOPE in the GUV’s membrane. The degree of deformation, i.e., the compactness (*C*_om_) of GUVs, under those conditions is determined. Fig 3 shows the deformation and compactness of the GUVs induced by 2.00 μg/mL NPs in the presence of 0, 2 and 5% PEG-DOPE in the membranes. Fig 3(A)–3(C) show the corresponding typical phase-contrast images of the deformation of PEG-DOPE/DOPC (0/100)-GUV, PEG-DOPE/DOPC (2/98)-GUV, and PEG-DOPE/DOPC (5/95)-GUV with the advancement of interaction times. Here, the shape of the GUVs is shown after every 10 min until 1 h observation. In all three cases, before the binding of NPs with GUV-membrane, that is, at *t* = 0 min, the vesicles were perfectly spherical in shape. After the interaction of NPs with the GUVs, the shape of the GUV gradually distorted.

The typical images indicate that at 60 min of interaction, the minimum and maximum deformation occurred in PEG-DOPE/DOPC (0/100)-GUV and PEG-DOPE/DOPC (5/95)-GUV, respectively. Among the above mentioned three categories of GUVs (based on PEG-DOPE concentration), after 10 min of interaction, for PEG-DOPE/DOPC (2/98)-GUV and PEG-DOPE/DOPC (5/95)-GUV, a little distortion is observable, whereas on and after 30 min, a significant deformation occurred in both cases and the GUVs are prolate-shaped ones. The time course of *C*_om_ of the same GUV is shown in Fig 3(D) that shows that *C*_om_ was 1.0 at *t* = 0 min for all three cases. At 60 min, the value of *C*_om_ is maximum (1.41) for PEG-DOPE/DOPC (5/95)-GUV and minimum (1.17) for PEG-DOPE/DOPC (0/100)-GUV. Fig 3(E) shows the time-course of average compactness, $C_{om}^{av}$, for all of the analyzed GUVs with different PEG-DOPE concentrations. We performed 2–4 independent experiments (i.e., *N* = 2–4) for each case where each independent experiments comprising a total number of 15–20 GUVs (i.e., *n* = 15–20). The error-bars indicate the SE of the $C_{om}^{av}$.

**Fig 4:**
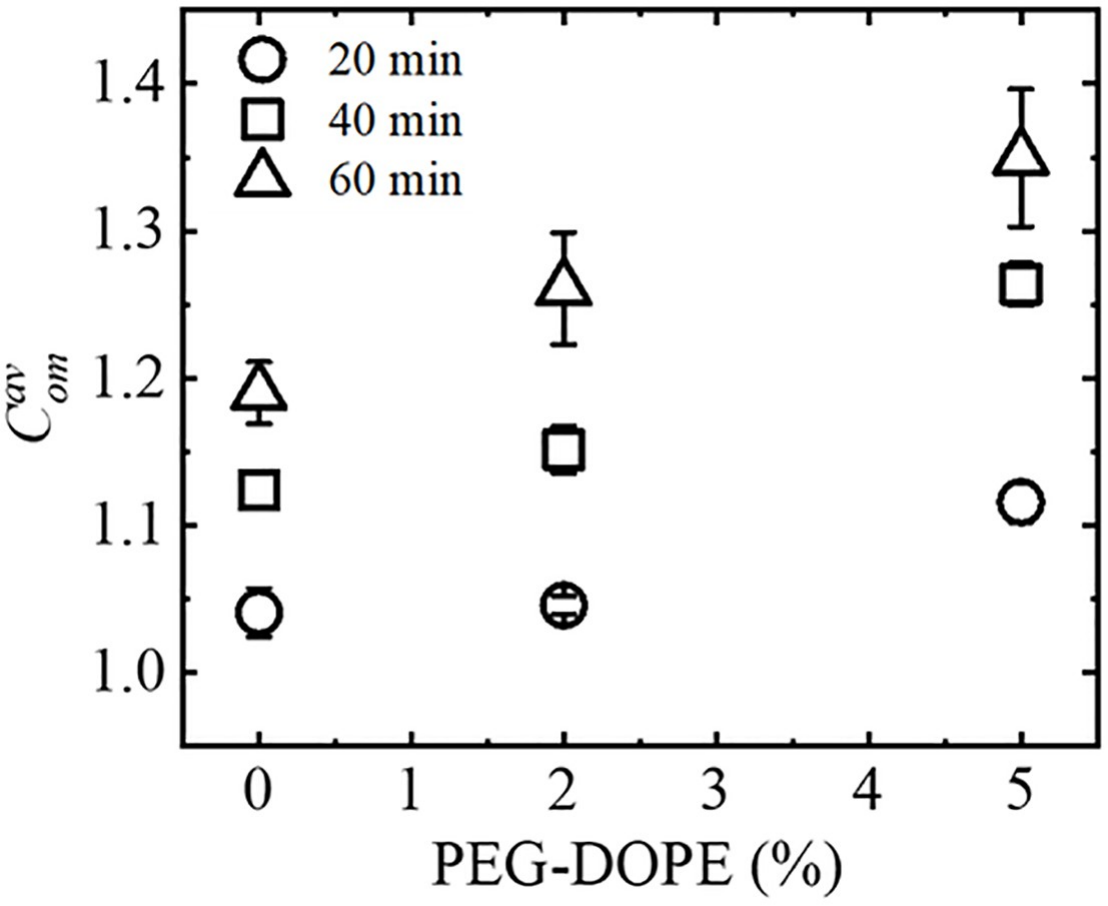
Dependence of average compactness with mole% of PEG-DOPE. The mole% of PEG-DOPE dependent average compactness at 20, 40, and 60 min of interaction of NPs with GUVs.

The values of the time dependent compactness and average compactness of PEG-DOPE/DOPC-GUVs for various PEG-DOPE (%) are presented in SI 1 Table in S1 File. The experiment on PEG-DOPE/DOPC (2/98)-GUV without leakage of encapsulating calcein is shown in section SI 3 Fig in S1 File. Fig 4 shows the dependence of $C_{om}^{av}$ (with SErr) on the mole% of PEG-DOPE in the GUV-membrane at 20, 40, and 60 min of interactions of the GUVs with NPs. It indicates that for each stage, the average compactness is maximum for PEG-DOPE/DOPC (5/95)-GUV and minimum for PEG-DOPE/DOPC (0/100)-GUV. For instance, at 60 min, the average compactness, $C_{om}^{av},$ for PEG-DOPE/DOPC (5/95)-GUVs was 1.35 ± 0.05, whereas for PEG-DOPE/DOPC (0/100)-GUVs, the average compactness was 1.19 ± 0.02. This result indicates that the NPs-induced deformation in PEG-DOPE/DOPC-GUVs increased with the increase of PEG-DOPE concentration in the membranes. Thus, PEG-DOPE plays an important role on the NPs-induced deformation in GUVs. The values of PEG-DOPE (%) dependent average compactness of PEG-DOPE/DOPC-GUVs at different time points are presented in SI 3 Table in S1 File.

**Fig 5:**
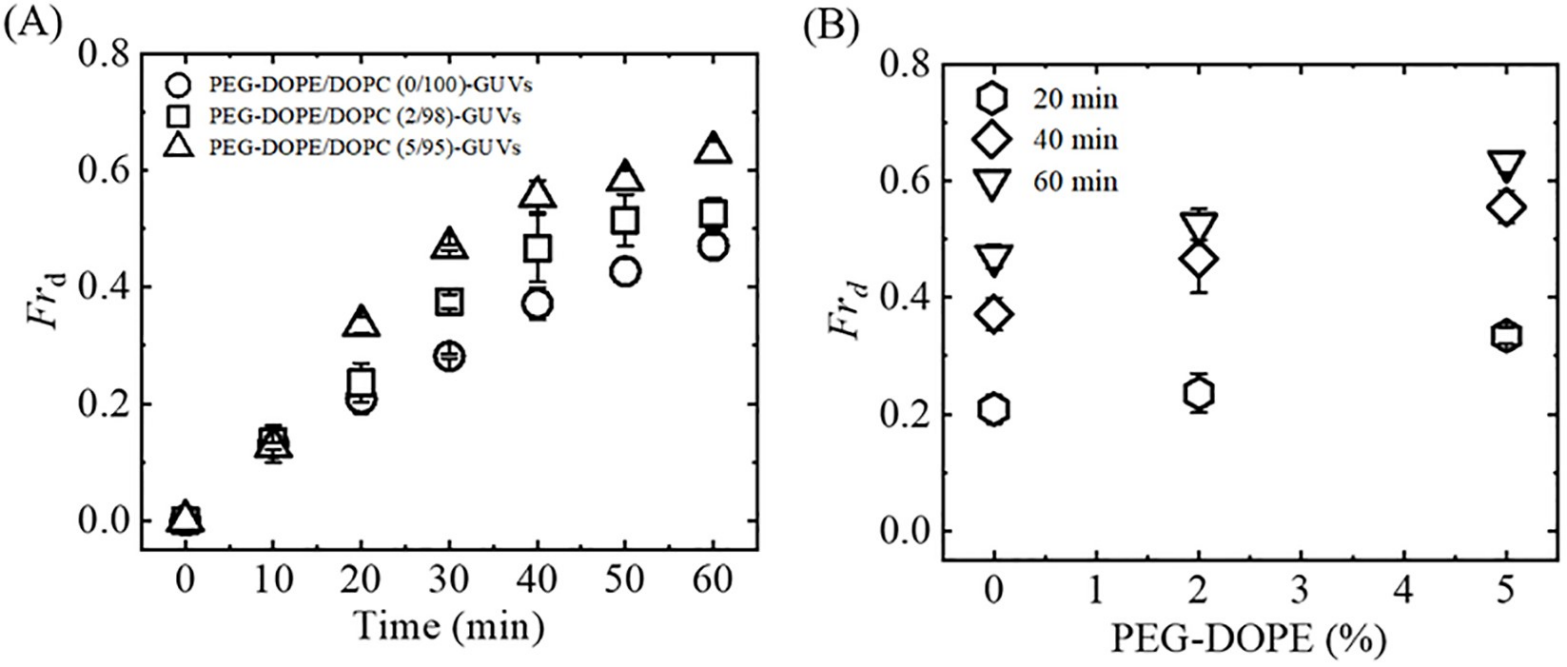
NPs-induced deformation of PEG-DOPE/DOPC-GUVs with various mole% of PEG-DOPE. (A) Time-course of the fraction of deformed GUVs (*Fr*_d_) for various mole% of PEG-DOPE in the membranes. (B) Dependence of *Fr*_d_ on mole% of PEG-DOPE at 20, 40, and 60 min of interaction of NPs with GUVs.

We also calculated the fraction of deformation (*Fr*_d_) of the GUVs under various experimental conditions. Fig 5 demonstrats the NPs-induced deformation of the of PEG-DOPE/DOPC-GUVs with various mole% of PEG-DOPE, where Fig 5(A) shows the time-course of the average *Fr*_d_ among all the experimented GUVs. We performed 2–4 independent experiments (i.e., *N* = 2–4) for each case where each independent experiments comprising a total number of 15–20 GUVs (i.e., *n* = 15–20). The error-bars indicate the standard error (SErr) of the *Fr*_d_. The trend-lines show that *Fr*_d_ increases with time for all categories of GUVs. Noteworthy, *Fr*_d_ is clearly maximum for PEG-DOPE/DOPC (5/95)-GUVs at 20 min and afterwards. Fig 5(B) shows the dependence of *Fr*_d_ on mole% of PEG-DOPE at different time of interactions (e.g., at 20, 40, and 60 min). This indicates that the *Fr*_d_ is highest for PEG-DOPE/DOPC (5/95)-GUVs and lowest for PEG-DOPE/ DOPC (0/100)-GUVs. For instance, at 60 min, the values of *Fr*_d_ for 0 and 5% PEG-DOPE were 0.47 ± 0.02 and 0.63 ± 0.02, respectively. Therefore, the results suggest that the presence of PEG-DOPE in the membranes enhances the NPs-induced deformation of GUVs. The values of the time dependent average *Fr*_d_ of PEG-DOPE/DOPC-GUVs for various PEG-DOPE (%) are presented in SI 4 Table in S1 File. In addition, the values of PEG-DOPE (%) dependent average *Fr*_d_ of PEG-DOPE/DOPC-GUVs at different time points are shown in SI 5 Table in S1 File.

### 3.2 Calcein leakage from PEG-DOPE/DOPC-GUVs due to the interaction of 2.00 μg/mL NPs

**Fig 6:**
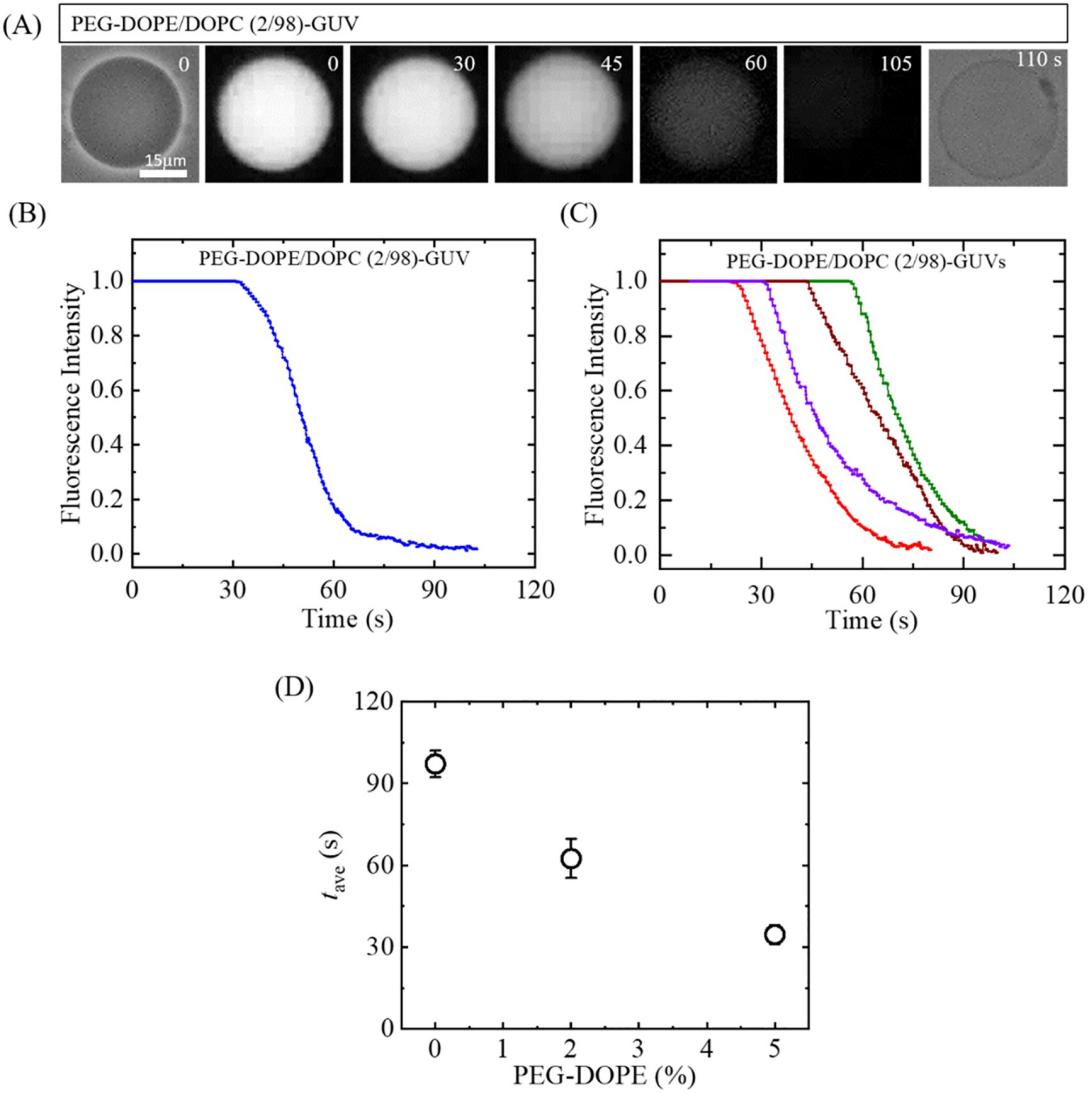
Leakage of calcein from the PEG-DOPE/DOPC-GUVs due to the interaction of 2.00 μg/mL NPs. (A) Interaction of NPs with PEG-DOPE/DOPC (2/98)-GUV containing 1 mM calcein and 0.10 M sucrose in a physiological condition. (i) and (iii) show the phase-contrast images of the GUV before and after the interaction of NPs, respectively. (ii) shows the fluorescence images of the GUV at various stage of interaction (interaction time is shown in second above each fluorescent image) with NPs. (B) The time-course of the change in normalized fluorescence intensity of GUV as shown in (A). (C) The time-course of the normalized fluorescence intensity for 4 different GUVs under the same condition as in (B). (D) Average time (*t*_ave_) of the onset of pore formation in GUVs under various mole% of PEG-DOPE in the membranes.

To examine whether there was poration in GUV-membrane due to the interaction of NPs, we investigated the calcein leakage from PEG-DOPE/DOPC-GUVs with the variation of PEG-DOPE concentration induced by 2.00 μg/mL NPs. Fig 6(A) shows a typical result on the interaction of NPs with a PEG-DOPE/DOPC (2/98)-GUV. The phase contrast image at *t* = 0 s shows that initially the contrast of the GUV is high (due to the difference in refractive index of sucrose and glucose present in the inside and outside solution of the GUV, respectively). The fluorescence microscopic image of the same GUV is shown at *t* = 0 s. The high fluorescence of the GUV-lumen is due to the presence of calcein inside the GUV. The fluorescence intensity within the GUV stays essentially consistent during the first 30 s after adding the NPs, and then the lumen intensity starts to decrease gradually (see Fig 6(A) & 6(B)), and eventually approaches to zero so that the GUV is not clearly distinguishable (at 105 s and afterwards) from the background using fluorescent microscopy. But, the corresponding phase-contrast microscopic view (at 110 s) shows that the spherical GUV exists, but the contrast of the GUV decreased greatly. This result indicates that the internal contents of the GUV, including calcein and sucrose, leaked out almost completely at 105 s. However, there is no increase in background fluorescence as the volume of the outer buffer is very large compared to that of leaked calcein probe.

In addition, the result suggests that the leakage of GUV’s internal contents, including calcein, occurred due to the formation of nanopores in the membrane. In several experiments, the use of various membrane-active agents (e.g., peptides, NPs, toxins) has been investigated to induce poration in the lipid membrane. This poration leads to the leakage or membrane-permeation of the encapsulated fluorescent material from the inside to the outside of vesicles [52–56]. The time-course of normalized fluorescence intensity of the GUV is shown in Fig 6(B). Fig 6(C) shows the normalized intensity profile for several typical GUVs. For each time profile, the onset of nanopores formation leading leakage of calcein solution is clearly revealed. We performed 2–4 independent experiments for each condition, where each independent experiment contained 15–20 GUVs.

The results indicate that the event of poration in GUV-membrane occurs in two stages; one is the adsorption of NPs on the membrane surface of the intact GUV, and the other is the formation of transmembrane nanopores through which the water-soluble fluorescent dye calcein leaks out from the inside of GUV. Thus, the NPs-induced poration in PEG-DOPE/DOPC-GUVs obeys two-state transition model. As the onset time of poration is different for different GUVs, so the NP-induced pore formation in GUVs is a stochastic phenomenon. Fig 6(D) shows the average time of the onset of pore formation, *t*_ave_ (± SErr), under various mole% of PEG-DOPE. With the increase of PEG-DOPE in the membranes, the *t*_ave_ decreases. The values of the PEG-DOPE (%) dependent average *t*_ave_ of PEG-DOPE/DOPC-GUVs are presented in SI 6 Table in S1 File. Therefore, the results of the calcein leakage experiment suggest that PEG-DOPE increases the NPs-induced pore formation in the GUV-membrane.

**Fig 7:**
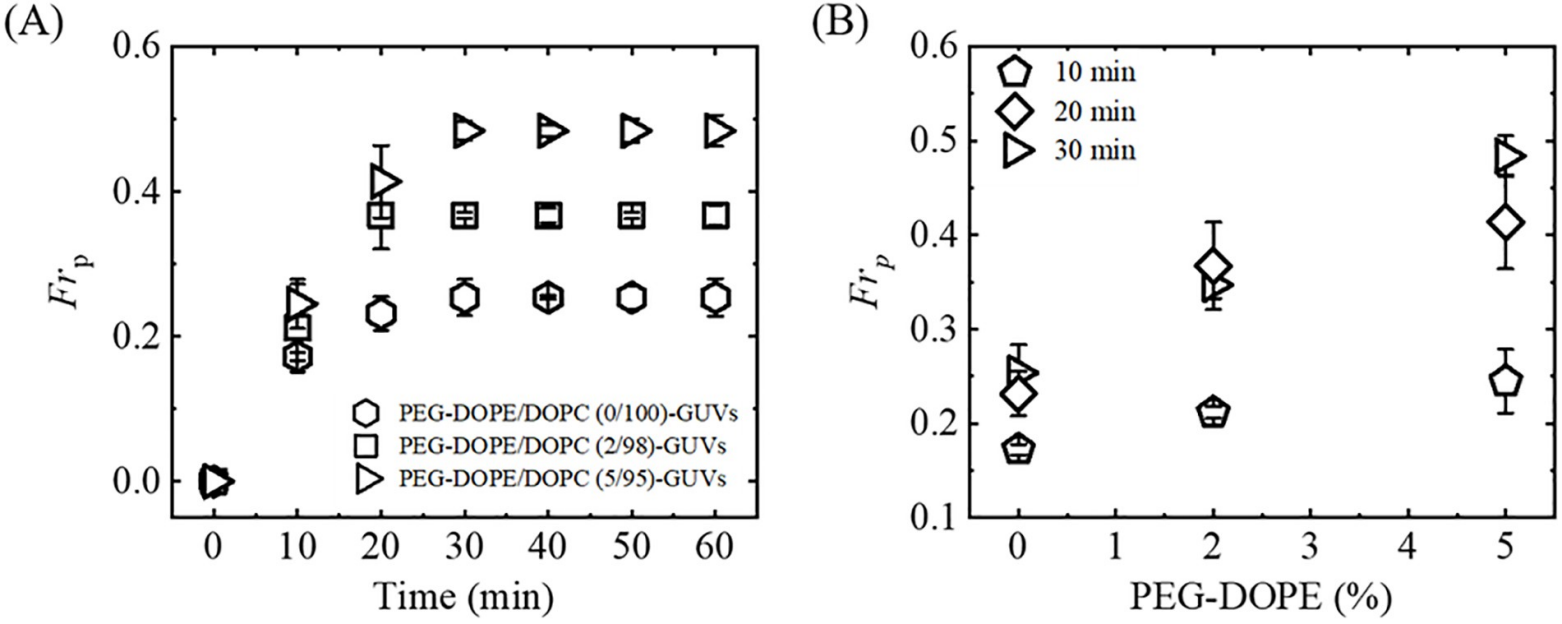
Time and PEG-DOPE (%) dependent fraction of pore formed GUVs (*Fr*_p_). (A) Time-course of the average fraction of pore-formed PEG-DOPE/DOPC-GUVs under various mole% of PEG-DOPE in the membranes. (B) Dependence of *Fr*_p_ on mole% of PEG-DOPE at 20, 40, and 60 min of interactions of NPs with GUVs.

Fig 7(A) shows the time-course of the average fraction of pore-formed GUVs, *Fr*_p_ (± SE), among all the examined GUVs (*N* = 2–4, *n* = 15–20) using 0, 2 and 5 mole% PEG-DOPE in DOPC-GUVs. The time-course of *Fr*_p_ indicates that the fraction of poration increased initially rapidly, then gradually and finally became nearly stable. The graph of dependence of *Fr*_p_ on mole% of PEG-DOPE indicates that the *Fr*_p_ increased with mole% of PEG-DOPE (Fig 7B). For example, the values of *Fr*_p_ (with SE) for 0 and 5% PEG-DOPE were 0.25 ± 0.02 and 0.48 ± 0.02, respectively. The values of the time-dependent average *Fr*_p_ of PEG-DOPE/DOPC-GUVs for various PEG-DOPE (%) are shown in SI 7 Table in S1 File. In addition, the values of the PEG-DOPE (%) dependent average *Fr*_p_ of PEG-DOPE/DOPC-GUVs at different time points are shown in SI 8 Table in S1 File.

**Fig 8:**
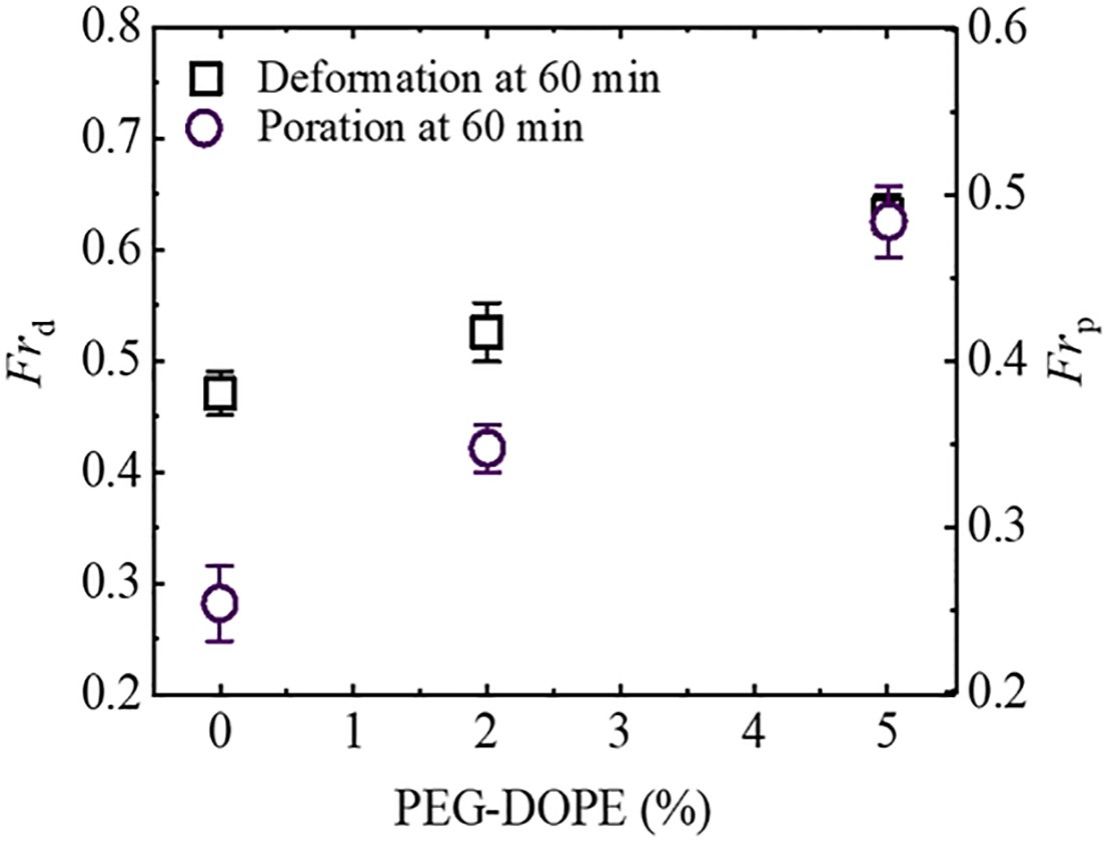
The mole% of PEG-DOPE dependent fraction of deformed (*Fr*_d_) and pore formed (*Fr*_p_) GUVs. Comparative analysis of the fraction of deformed GUVs and fraction of pore-formed GUVs at 60 min of interaction of NPs with GUVs under various mole% of PEG-DOPE.

It is worthy to mention the comparative analysis of the fraction of deformed GUVs and fraction of pore-formed GUVs at *t* = 60 min of interactions of NPs with GUVs under various mole% of PEG-DOPE (Fig 8). Here, the data-points clearly indicates that both deformation and poration enhanced with the increase of PEG-DOPE in the membranes. However, the fraction of deformation is relatively higher than the fraction of poration for each mole% PEG-DOPE in the membranes. The comparison of PEG-DOPE (%) dependent average *Fr*_d_ and *Fr*_p_ of PEG-DOPE/DOPC-GUVs is shown in SI 9 Table in S1 File.

## 4. Discussion

In this study, we examined the effect of PEG-DOPE on NPs-induced deformation and poration of the PEG-DOPE/DOPC-GUVs under physiological conditions. We found that NPs-induced deformation and poration of GUVs comprising PEG-DOPE and DOPC, where the concentration of PEG-DOPE affected both the pore formation and deformation. It should be mentioned that antimicrobial peptide magainin 2 induces similar sort of nanopore formation in biomembranes and lipid bilayers of GUVs under physiological condition [29, 54, 57]. However, the most emphasizing part of this report is that the fraction of poration (*Fr*_p_), average compactness of the GUVs ($C_{om}^{av}$) and the fraction of deformation of the GUVs (*Fr*_d_) induced by the NPs increases with the increase of mole% of PEG-DOPE in the membranes.

**Fig 9:**
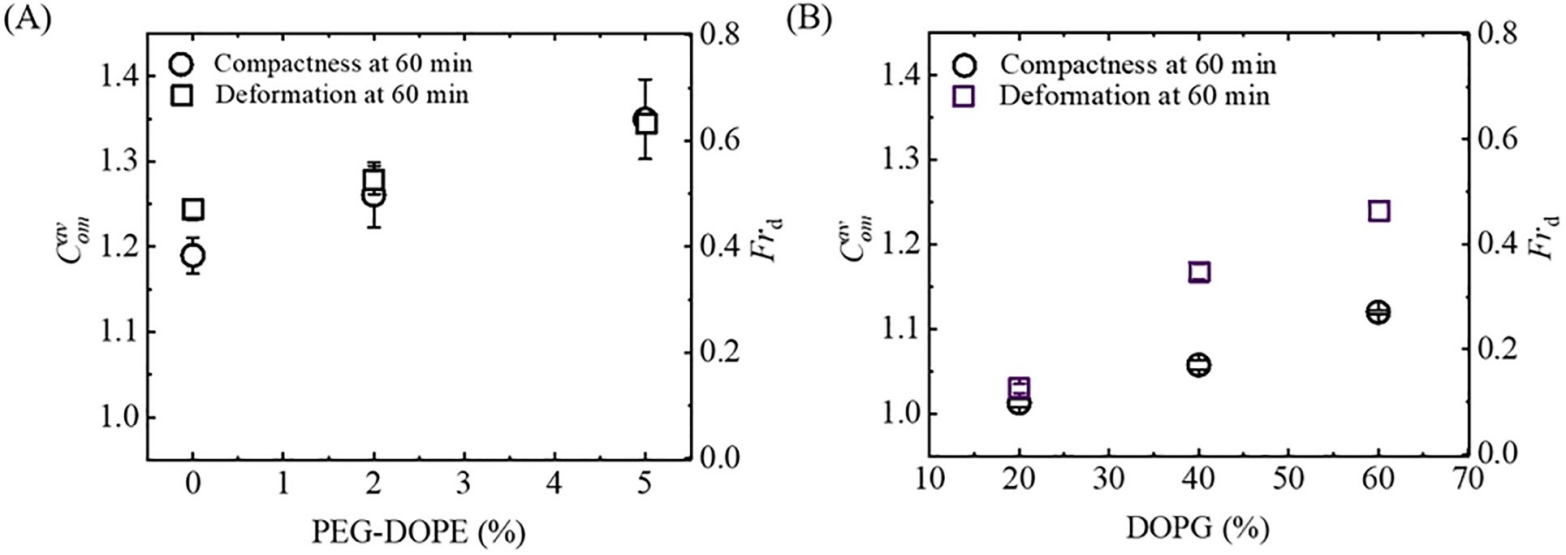
Average compactness ($C_{om}^{av}$) and fraction of deformation (*Fr*_d_) under various mole% of PEG-DOPE and DOPG. (A) Comparison between the $C_{om}^{av}$ and *Fr*_d_ under the variation of- (A) mole% of PEG-DOPE in PEG-DOPE/DOPC-GUVs and (B) mole% of DOPG in DOPG/DOPC-GUVs.

At first, we want to discuss the results examining the effect of PEG-DOPE-grafted lipid on NPs-induced deformation of the vesicles. Regarding this, the parameters *Fr*_d_ and $C_{om}^{av}$ have been used to quantify the degree of deformation and percentage of deformation. It is worthy to mention here that the values of $C_{om}^{av}$ and *Fr*_d_ increase with the increase of mole% PEG-DOPE in the membrane. We can mention a particular case, where for 5% PEG-DOPE in the membrane, the values of $C_{om}^{av}$ and *Fr*_d_ after 60 min of interaction are correspondingly 1.35 ± 0.05 and 0.63 ± 0.02 (see Fig 9A). The results of this report can be compared with the numerical values of the same parameters (i.e., $C_{om}^{av}$ and *Fr*_d_) of DOPG/DOPC-GUVs for various concentrations of DOPG [49]. At 60 min of interaction of the same concentration of NPs (i.e., 2.00 μg/mL) with DOPG/DOPC-GUVs, $C_{om}^{av}$ and *Fr*_d_ increased with the increase of mole% DOPG in DOPG/DOPC-GUVs (see Fig 9B). But, for 5% PEG-DOPE, both of $C_{om}^{av}$ and *Fr*_d_ are much larger in PEG-DOPE/DOPC-GUVs than that of DOPG/DOPC-GUVs even for 60% DOPG. Thus, the presence of polymer-grafted-lipid greatly influences the deformation of the vesicles.

Regarding the perturbation of membrane stability induced by NPs, previously it was reported that the adsorption of anionic NPs changes the local phase state of a phospholipid bilayer. Local gelation in a fluid bilayer was generated by the binding of anionic (0.91 e^-^/nm^2^) NPs with DOPC membrane [58]. The deformation of the PEG-DOPE/DOPC-GUVs induced by the anionic NPs can be explained combinedly with the aid of the bilayer-coupling model (BC model) and the area-difference elasticity model (ADE model). According to the BC model [59, 60], equilibrium shapes of phospholipid vesicles are assumed to correspond to the lowest possible elastic energy of the closed membrane bilayer.

It is worthy to mention that GUVs can form several stable structures varying shapes, and all of these stable structures hold a particular free energy, given by *E*_i_. This free energy is the measure how stable a vesicle shape is. Suppose, a nearly-spherical shaped GUV has the lowest free energy, given by *E*_sph_. On the other hand, the probability of existence of a stable non-spherical vesicle is much smaller than that of spherical one as *E*_def_ > > *E*_sph_, where *E*_def_ is the free energy of the non-spherical or deformed vesicle. Consequently, in the physiological condition when the vesicles are not perturbed by any external agent (e.g., shrinkage of vesicles due to negative osmotic pressure), the probability of finding a spherical GUV is tends to the unity and the probability of finding the non-spherical one is nearly zero. The fraction of deformed GUVs (*Fr*_d_) in a suspension of vesicles is inversely proportional to the deviation of free energy of a deformed GUV from the spherical one, as follows [49]:

$${Fr}_{d}\propto \frac{1}{E_{def}-E_{sph}}\approx \frac{1}{E_{def}}(1+\frac{E_{sph}}{E_{def}})$$ (2)

Eq (2) indicates that for the shape transformation of a spherical vesicle to a non-spherical one, the GUV must overcome an energy barrier (*E*_bar_) given by, *E*_bar_ = *E*_def_ − *E*_sph_, that determines the rate of transformation of shapes of the GUVs. The large polymer, PEG, attached to the headgroup of DOPE phospholipid accelerates the deformation and poration events in GUVs induced by the NPs as per the experimental results (see Section 3). We infer that a large entropy that is developed in the system of membrane-water interface due to the long chain of PEG polymer continuously perturb the lipid membrane stability. The effect of PEG-chain on the membrane permeation of the liposome’s internal contents was reported previously [2, 44]. Mahendra and colleagues reported that with the increase of mole percentage of PEG-lipid (they used DSPE-PEG) in vesicles, the bending modulus of the membrane and permeability of water of the lipid bilayer increases, but the change in elastic area compressibility constant of the bilayer was trivial [2].

The structure of GUVs is identified by the closed bilayer elastic energy (*W*el) minimization. This energy only considers the membrane bending energy (*W*ben), not the elastic stretching of monolayers. For a given area and volume of GUVs, the area distinction among the two single-layers of a bilayer determines the minimum elastic energy [59, 61, 62]. Later, area difference elasticity (ADE) model was considered to explain the change of shape of vesicles [63, 64]. The area of each monolayer of the lipid bilayer in the ADE model is not set to be the symmetrical one but can be extended elastically to enhance the membranes’ non-linear bending energy. In regards the two-dimensional membrane elasticity, the primary model of membrane elasticity gives the bending energy of the membrane [65]. The elastic energy of the closed membrane bilayer (*W*_el_) is equal to the sum of the bending energy of the membrane (*W*_ben_) and the energy of the relative stretching of the outer monolayer of the membrane bilayer (*W*_r_), as described below:

$$W_{el}=W_{ben}+W_{r}=\left(\frac{k_{l}}{2}\right)\int {\left(C_{1}+C_{2}\right)}^{2}dA+\left(\frac{k_{r}}{2A_{0}h^{2}}\right)(\Delta A-\Delta A_{0}{)}^{2}$$ (3)

where, *k*l is the local bending modulus of membrane and *k*r is the relative (non-local) bending modulus of the membrane. The integration is performed over the so-called neutral surface of membrane. In the integration part, the parameters *C*1 and *C*2 indicate the principal membrane curvatures at a point of a monolayer giving the maximum and minimum values of a curvature. Noteworthy, *C*1 and *C*2 are positive for a spherical shaped body and the unit normal vector of the surface points inward. At stretched state of the membrane, the difference between the surface area of the outer monolayer and the inner monolayer is Δ*A* = *A*_out_ − *A*_in_, where *A*_out_ and *A*_in_ are the surface area of the stretched outer monolayer and inner monolayer, respectively. Similarly, the difference between the surface area at equilibrium is Δ*A*_o_ = *A*_out,0_ − *A*_in,0_. The parameter *h* is the separation between the outer monolayer and the inner monolayer in a neutral membrane surface. According to the ADE model, the shape of the GUVs is governed by the minimization of the elastic energy of the membrane that is obtained at constant membrane area and vesicle volume. If the area difference of the GUV-membrane at equilibrium state and stretched state are Δ*A*_0_ and Δ*A*, respectively, then for a constant volume of GUVs, shape change of the vesicles is controlled by the term (Δ*A* − Δ*A*_0_)^2^ of Eq (3). Based on this discussion, it can be inferred that when NPs adsorb onto the outer monolayer of lipid bilayer of GUVs, it induces an area mismatch between the outer and inner monolayers. Consequently, this area mismatch alters the free energy of the vesicle. In order to reduce their free energy and achieve a state of minimum energy (so that the vesicle obtain more stability than the earlier shape), the GUVs undergo a change in their shape, transitioning from a spherical form to adopt alternative shapes.

Furthermore, we focus on the discussion of the results examining the effect of PEG-DOPE on NPs-induced poration of the vesicles. Regarding this, the parameter of fraction of pore-formed (leaked) GUVs, *Fr*_p_, was used to quantify the percentage of pore formation in GUVs among all examined GUVs. It is worthy to mention here that the value of *Fr*_p_ increases with the increase of mole% of PEG-DOPE in the membrane. We can mention a particular case, the fraction of poration (*Fr*_p_) at 60 min of interaction (for details, see Fig 8) for 0% and 5% PEG-DOPE were 0.25 ± 0.02 and 0.48 ± 0.02, respectively. The electrostatic attraction between anionic NPs and N+ (i.e., repulsion between NPs and P-) and the intra-membrane electrostatic effect for PEG-DOPE (here, DOPE segment in DOPE-PEG becomes negatively charged in physiological environment) are the two interaction effects present in the case of PEG-DOPE/DOPC-GUVs. The fraction of deformed GUVs increased as the mole% of PEG-DOPE increased in the membranes of GUVs. It should be mentioned here, the inner monolayer of a GUV is stretched when a cationic antimicrobial peptide (e.g., magainin 2) binds to the outer monolayer, increasing the area of the bilayer [29, 54]. The stretching of lipid bilayer or lateral stress inducing on the membrane by any external forces causes the creation of pore leading rupture of the vesicles [32, 35]. Thus, stretching of the membrane induces the lateral tension in the inner monolayer, which induces pore formation and development in the membranes. It is found experimentally that the pore formation time happened earlier for PEG-DOPE/DOPC (5/95)-GUVs compared to that of PEG-DOPE/DOPC (0/100)-GUVs (see Fig 6D). Due to the interaction of anionic NPs with GUVs, the formation of pores in GUVs has been investigated in several experiments [53, 55]. We can reasonably consider that the binding of NPs in the outer monolayer causes the inner monolayer to stretch, which results formation of pores in the membranes.

**Fig 10:**
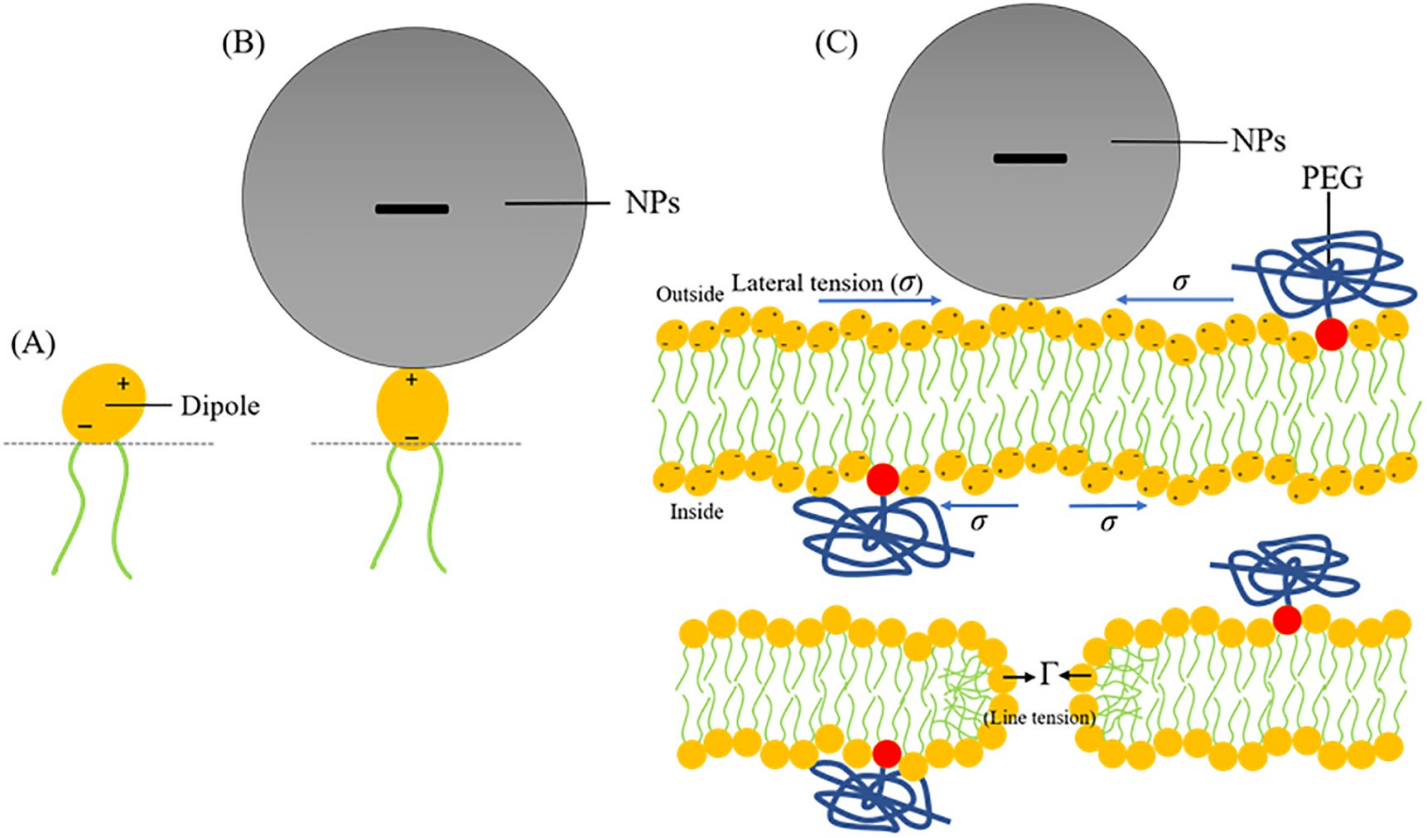
A possible mechanism of the binding of NPs with PEG-DOPE/DOPC-GUVs that results poration in the membranes. (A) Electric dipole formed in the head-group of zwitterionic (charge-neutral) DOPC phospholipid between the negatively charged phosphate group (P^−^) and positively charged choline group (N^+^). Initially, the dipole moment vector is aligned with an acute angle to the bilayer-surface. (B) The adsorbed anionic NPs (Gray sphere) is attracted with the N^+^ terminus of P^−^–N^+^ dipole due to the electrostatic interaction changing the alignment of the dipole. (C) Due to the adsorption of the NPs on the outside of bilayer-surface, lateral tension (*σ*) is generated in both monolayers of the bilayer. Nanopore formation occurs (not visible using optical microscope) in the membrane. Nanopore closes due to the large line tension (Γ) at the pore-rim.

Based on the experimental conditions and pore formation event observed in the current project, here we propose a hypothesis on the adsorption of the anionic NPs on the outer monolayer of lipid bilayers which results poration in GUV-membrane comprising PEG-DOPE and DOPC (see Fig 10). Electric dipoles are formed in the head-groups of charge-neutral DOPC phospholipid between the negatively charged phosphate group (P^−^) and positively charged choline group (N^+^). Initially, the dipole moment vector is aligned with an acute angle to the bilayer-surface. The adsorbed anionic NPs (in Fig 10A, a Gray sphere depicts the adsorbed NPs on the membrane) is electrically attracted with the N^+^ terminus of the P^−^–N^+^ dipole due to the electrostatic attraction that changes the alignment of the dipole significantly (Fig 10B). We consider that, due to the adsorption of the NPs on the outer monolayer, a lateral tension (*σ*) is generated both in the outer and inner monolayers of the bilayer, but the direction of *σ* is opposite.

As a result of local rarefaction of lipid molecules is generated, nanometer-scale pores (i.e., nanopores) formation occurs (which is not visible using optical microscope) in the membrane through which internal contents of the GUVs, including the fluorescent probe, leaked out. But, the nanopores cannot grow further to micrometer-scale pores (i.e., micropores) that would be visible through optical microscope due to the large line-tension (Γ) at the pore-rim which dominates over the lateral tension *σ* to close the pore afterward. Therefore, the final phase-contrast view of the leaked-GUVs show a spherical-shaped GUV similar to that of intact GUV with only difference is the significant decrease of the phase-contrast of the GUV due to the leakage of sucrose solution through the nanopores.

## 5. Conclusions

In this study, we investigate the influence of PEG-DOPE on the deformation and poration of PEG-DOPE/DOPC-GUVs triggered by the anionic magnetite NPs. Specifically, using a concentration of 2.00 μg/mL NPs, the fraction of deformation of the vesicles and the leakage of calcein dye from the vesicles were examined and the corresponding mechanisms are proposed. We revealed that with the increase of PEG-DOPE concentration in the membrane, the fraction of deformation and the fraction of poration of the experimented GUVs increases, whereas the average time of poration decreases. This indicates, PEG-DOPE promotes NPs-induced deformation and poration of the vesicles. In the deformation experiments, the parameter compactness adapted to measure the degree of deformation of the GUVs indicates that following the addition of NPs in the suspension of spherical shaped GUVs containing various mole-percentage of PEG-DOPE, the value of compactness increases with time. The result relating average compactness and PEG-DOPE (%) indicates, the higher the concentration of PEG-DOPE in the membrane, the greater value of average compactness. Noteworthy, the value of average compactness for 5%PEG-DOPE is greater than that of 60%DOPG. Therefore, the effect of PEG-DOPE on NPs-induced deformation of GUVs is greater than that of DOPG. Also, following the addition of NPs in the suspension of vesicles, the fluorescence intensity of the GUV-lumen remained constant for a while and then decreased gradually to become zero with time. Thus, poration in GUV-membrane occurs in two stages: first, adsorption of NPs on the membrane surface, and then, formation of transmembrane nanopores through which membrane permeation of water-soluble fluorescent dye calcein occurs. To the best of our knowledge, this study is the first experimental investigation determining the effect of PEG-grafted phospholipid on anionic magnetite NPs-induced deformation and poration in lipid membranes. It is expected that this research could help to understand the effects of polymer-grafted phospholipids on the NPs-induced deformation and poration in real cells.

## Supporting information

S1 File (PDF)
