# Supplementary material for: Effects of polyethylene glycol-grafted phospholipid on the anionic magnetite nanoparticles-induced deformation and poration in giant lipid vesicles
Source: PLoS One. 2023 Jul 31;18(7):e0289087. doi: 10.1371/journal.pone.0289087 (PMC10389724; doi:10.1371/journal.pone.0289087)
Supplement: S1 File — (PDF) [file pone.0289087.s001.pdf]

Supplementary Information (SI)

Effects of polyethylene glycol-grafted phospholipid on the anionic magnetite nanoparticles-induced deformation and poration in giant lipid vesicles

Mohammad Abu Sayem Karal, Sharmin Sultana, Md. Masum Billah, Md. Moniruzzaman, Md. Abdul Wadud, and RC Gosh

SI 1 Compactness measurement

Fig SI 1 presents an illustration demonstrating the measurement of compactness (C_{om}) for both a perfectly spherical-shaped GUV and a deformed GUV. The analysis involved selecting the rim of the images, indicated by the white dotted line, and extracting the corresponding perimeter (P) and cross-sectional area (S_{cr}) of the GUV using MATLAB's image processing toolbox. Subsequently, the values of C_{om} were calculated using Eq (1). For Fig SI 1(A), the obtained C_{om} value is 1.0, indicating a perfect spherical shape, while Fig SI 1(B) exhibits a higher C_{om} value, indicating a deformed shape.

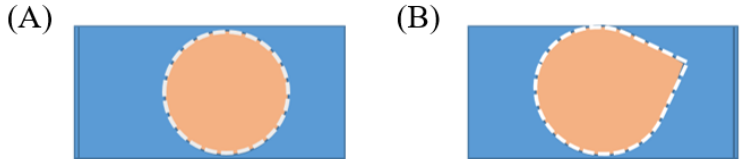

Fig SI 1. Illustration of measuring the compactness of a 'single GUV'. (A) A perfectly spherical shaped GUV. (B) Deformed GUV

The parameter C_{om} can be related with the real physical parameters of the system, particularly, it can be related with the surface area (S_t) of the deformed GUV at time t. Considering a case where a deformed GUV gained the geometry of ellipsoid which was spherical in shape at t = 0 s (before interacting NPs), one can obtain the following relation between C_{om} and S_t [1]:

$$S_{\mathrm{t}} \approx S_0[1 + 1.78\sqrt{(C_{\mathrm{om}} - 1)}] \qquad (1)$$

where S_0 surface area of the spherical-shaped (non-distorted) GUV before the interaction of NPs. Both C_{om} and S_t/S_0 increases with time for both charged and neutral membranes as obtained in our previous investigation [1].

SI 2 Microchamber

The microchamber, as shown in Fig SI 2, comprised a glass slide, a silicon-rubber spacer, and a cover slip, forming a U-shaped structure.

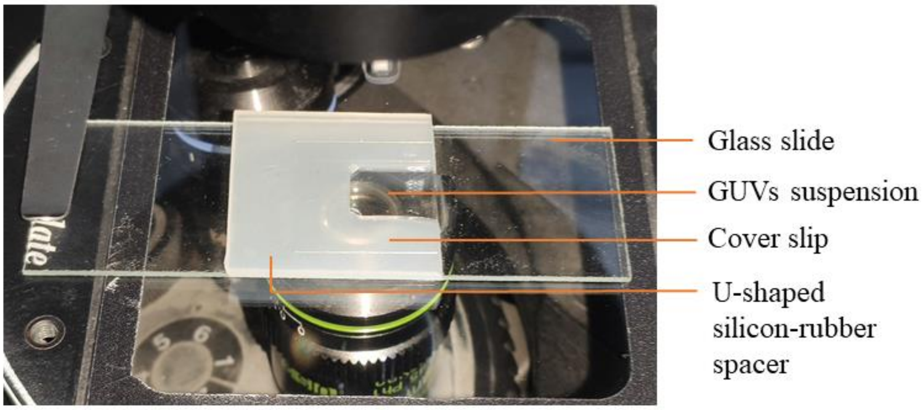

Fig SI 2. A photo of microchamber used for the observations of GUVs.

SI 3 Experiment on PEG-DOPE/DOPC (2/98)-GUV without leakage of encapsulating calcein

A controlled experiment was conducted using PEG-DOPE/DOPC (2/98)-GUV containing the fluorescent probe calcein. The results of this experiment are depicted in Fig SI 3(A) in the absence of NPs. Each image in the figure corresponds to a specific time of observation, as indicated by the number on the GUV images. In the first image of Fig SI 3(A), a phase contrast image of the GUV is shown. The inside and outside of the GUV exhibit a high contrast due to the differences in refractive indices resulting from the presence of sucrose and glucose. This image indicates the intact state of the GUV. The second to fifth images in Fig SI 3(A) display fluorescent images of the same GUV, with no observable loss of fluorescence intensity throughout the entire duration (0 –120 s) of the observation. The last image in the series represents the phase contrast image of the same GUV captured at 130 s. The contrast between the inside and outside of the GUV remains consistent at 130 s, similar to the observation at 0 s. Photobleaching of the calcein probe was not observed during the experiment, as two ND filters were utilized in the fluorescence microscope, as mentioned in section 2.5. This ensured the prevention of excessive exposure to light. Fig SI 3(B) depicts the corresponding time-dependent fluorescence intensity of the GUV shown in Fig SI 3(A). The fluorescence intensity inside the GUV lumen remains constant throughout the entire observation period. The proper consideration of photobleaching effect is essential for obtaining reliable data and drawing accurate conclusions about biomacromolecular interactions. A quantitative assessment of these factors and their implications have been assessed in a recent report [2].

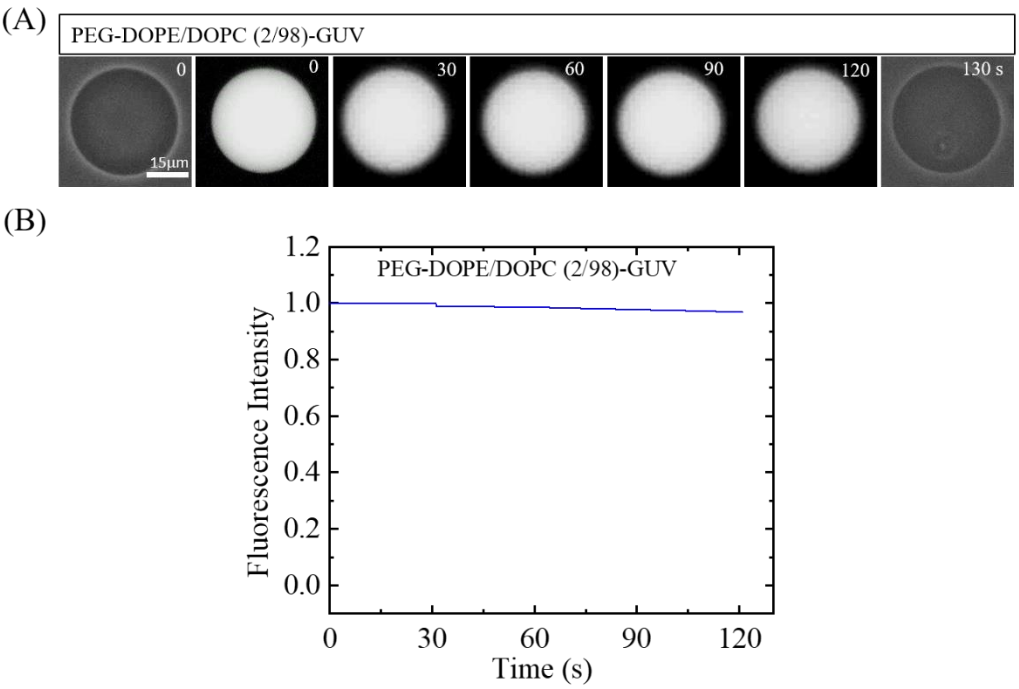

Fig SI 3. Experiment on PEG-DOPE/DOPC (2/98)-GUV without leakage of encapsulating calcein in the absence of NPs. (A) Phase contrast and fluorescent image of a GUV. (B) The time course of the florescence intensity of the GUV as shown in (A).

Table SI 1. Time dependent compactness (C_{om}) of PEG-DOPE/DOPC-GUVs for various PEG-DOPE corresponding to Fig 3(D).

Time (min)	PEG-DOPE/DOPC (0/100)	PEG-DOPE/DOPC (2/98)	PEG-DOPE/DOPC (5/95)
	C_{om}	C_{om}	C_{om}
0	1.0	1.0	1.0
5	1.00223	1.01070	1.03431
10	1.01562	1.01649	1.08425
15	1.03498	1.02128	1.13482
20	1.05758	1.03239	1.16620
25	1.07309	1.04582	1.19739
30	1.08623	1.07647	1.21884
35	1.09170	1.10457	1.23838
40	1.10481	1.14182	1.28593
45	1.11129	1.17457	1.30936
50	1.12538	1.21542	1.33178
55	1.14504	1.24792	1.37164
60	1.16856	1.26464	1.41495

Table SI 2. Time dependent average compactness (C_{om}^{av}) of PEG-DOPE/DOPC-GUVs for various PEG-DOPE corresponding to Fig 3(E).

Time (min)	PEG-DOPE/DOPC (0/100)		PEG-DOPE/DOPC (2/98)		PEG-DOPE/DOPC (5/95)	
	C_{om}^{av}	±SErr	C_{om}^{av}	±SErr	C_{om}^{av}	±SErr
0	1.0	0	1.0	0	1.0	0
5	1.00844	0.00186	1.02006	0.00088	1.01704	0.00607
10	1.01273	0.00123	1.02706	0.00272	1.05473	0.00918
15	1.02386	0.01056	1.03492	0.00348	1.08816	0.01966
20	1.04045	0.01647	1.04531	0.00631	1.11540	0.01343
25	1.06469	0.01579	1.05739	0.00859	1.16626	0.01792
30	1.09427	0.01208	1.07832	0.01093	1.21149	0.01486
35	1.11608	0.01235	1.10919	0.01726	1.22800	0.01045
40	1.12356	0.01246	1.15111	0.01506	1.26377	0.01457
45	1.13812	0.01237	1.18854	0.02163	1.29274	0.00664
50	1.14608	0.01281	1.21345	0.02577	1.30855	0.01528
55	1.16829	0.01911	1.23449	0.02719	1.32092	0.02769
60	1.18974	0.02111	1.26069	0.03818	1.34948	0.04672

Table SI 3. PEG-DOPE dependent average compactness (C_{om}^{av}) of PEG-DOPE/DOPC-GUVs at different time points corresponding to Fig 4.

Time (min)	PEG-DOPE (%)	C_{om}^{av}	±SErr
20	0	1.04045	0.01647
	2	1.04531	0.00631
	5	1.11540	0.01343
40	0	1.12356	0.01246
	2	1.15111	0.01606
	5	1.26377	0.01457
60	0	1.18974	0.02111
	2	1.26069	0.03818
	5	1.34948	0.04672

Table SI 4. Time dependent average fraction of deformation (Fr_d) of PEG-DOPE/DOPC-GUVs for various PEG-DOPE corresponding to Fig 5(A).

Time (min)	PEG-DOPE/DOPC (0/100)		PEG-DOPE/DOPC (2/98)		PEG-DOPE/DOPC (5/95)	
	Fr_d	±SErr	Fr_d	±SErr	Fr_d	±SErr
0	0	0	0	0	0	0
10	0.13173	0.03173	0.13627	0.00935	0.12738	0.00595
20	0.20882	0.02451	0.23615	0.03333	0.33460	0.01389
30	0.28143	0.00387	0.37506	0.01234	0.46739	0.00483
40	0.37177	0.02766	0.46657	0.05819	0.55524	0.02746
50	0.42719	0.02193	0.51496	0.04417	0.58333	0.01667
60	0.47087	0.01961	0.52556	0.02640	0.63185	0.01727

Table SI 5. PEG-DOPE dependent average fraction of deformation (Fr_d) of PEG-DOPE/DOPC-GUVs at different time points corresponding to Fig 5(B).

Time (min)	PEG-DOPE (%)	Fr_d	±SErr
20	0	0.20882	0.02451
	2	0.23615	0.03333
	5	0.33460	0.01389
40	0	0.37177	0.02766
	2	0.46657	0.05819
	5	0.55524	0.02746
60	0	0.47087	0.01961
	2	0.52556	0.02640
	5	0.63185	0.01727

Table SI 6. PEG-DOPE dependent average poration time (t_{ave}) of PEG-DOPE/DOPC-GUVs corresponding to Fig 6(D).

PEG-DOPE (%)	Poration time, t_{ave} (s)	
	Average	±SErr
0	97.2	4.9
2	62.5	7.2
5	34.6	3.4

Table SI 7. Time dependent average fraction of poration (Fr_p) of PEG-DOPE/DOPC-GUVs for various PEG-DOPE corresponding to Fig 7(A).

Time (min)	PEG-DOPE/DOPC (0/100)		PEG-DOPE/DOPC (2/98)		PEG-DOPE/DOPC (5/95)	
	Fr_p	±SErr	Fr_p	±SErr	Fr_p	±SErr
0	0	0	0	0	0	0
10	0.17228	0.00561	0.21164	0.06061	0.24484	0.03373
20	0.23162	0.02328	0.36721	0.04626	0.41394	0.05000
30	0.25380	0.02504	0.36721	0.00417	0.48394	0.01341
40	0.25380	0.00241	0.36721	0.01006	0.48394	0.00871
50	0.25380	0.01579	0.36721	0.00431	0.48394	0.01667
60	0.25380	0.02591	0.36721	0.01435	0.48394	0.02151

Table SI 8. PEG-DOPE dependent average fraction of poration (Fr_p) of PEG-DOPE/DOPC-GUVs at different time points corresponding to Fig 7(B).

Time (min)	PEG-DOPE (%)	Fr_p	±SErr
20	0	0.17228	0.00561
	2	0.21164	0.00606
	5	0.24484	0.03373
40	0	0.23162	0.02328
	2	0.36721	0.04626
	5	0.41394	0.05000
60	0	0.25380	0.02961
	2	0.34721	0.01435
	5	0.48394	0.02151

Table SI 9. Comparison of PEG-DOPE dependent average fraction of deformation (Fr_d) and fraction of poration (Fr_p) of PEG-DOPE/DOPC-GUVs corresponding to Fig 8.

PEG (%)	Fr_d		Fr_p	
	Average	±SErr	Average	±SErr
0	0.47087	0.01961	0.25380	0.02261
2	0.52556	0.02640	0.34721	0.01435
5	0.63185	0.01727	0.48394	0.02151

References

1. Karal MAS, Ahammed S, Levadny V, Belaya M, Ahamed MK, Ahmed M, et al. Deformation and poration of giant unilamellar vesicles induced by anionic nanoparticles. Chem Phys Lipids. 2020;230: 104916. doi:10.1016/j.chemphyslip.2020.104916

2. Park S, Jackman JA, Cho N-J. Quantitative accounting of dye leakage and photobleaching in single lipid vesicle measurements: Implications for biomacromolecular interaction analysis. Coll Surf B: Biointerf. 2019;182: 110338. doi:10.1016/j.colsurfb.2019.06.067
